# Anabolic Function Downstream of TOR Controls Trade-offs Between Longevity and Reproduction at the Level of Specific Tissues *in C. elegans*

**DOI:** 10.3389/fragi.2021.725068

**Published:** 2021-09-10

**Authors:** Amber C. Howard, Dilawar Mir, Santina Snow, Jordan Horrocks, Hussein Sayed, Zhengxin Ma, Aric N. Rogers

**Affiliations:** 1 Mount Desert Island Biological Laboratory, Davis Center for Regenerative Biology and Medicine, Bar Harbor, ME, United States,; 2 Department of Natural Sciences, Middle Georgia State University, Cochran, GA, United States,; 3 Department of Oncology, School of Medicine and Public Health, University of Wisconsin, Madison, WI, United States

**Keywords:** aging, adaptive response, mRNA translation, cell non-autonomous, lifespan, reproduction, trade-offs, optimal foraging theory

## Abstract

As the most energetically expensive cellular process, translation must be finely tuned to environmental conditions. Dietary restriction attenuates signaling through the nutrient sensing mTOR pathway, which reduces translation and redirects resources to preserve the soma. These responses are associated with increased lifespan but also anabolic impairment, phenotypes also observed when translation is genetically suppressed. Here, we restricted translation downstream of mTOR separately in major tissues in *C. elegans* to better understand their roles in systemic adaptation and whether consequences to anabolic impairment were separable from positive effects on lifespan. Lowering translation in neurons, hypodermis, or germline tissue led to increased lifespan under well-fed conditions and improved survival upon withdrawal of food, indicating that these are key tissues coordinating enhanced survival when protein synthesis is reduced. Surprisingly, lowering translation in body muscle during development shortened lifespan while accelerating and increasing reproduction, a reversal of phenotypic trade-offs associated with systemic translation suppression. Suppressing mTORC1 selectively in body muscle also increased reproduction while slowing motility during development. In nature, this may be indicative of reduced energy expenditure related to foraging, acting as a “GO!” signal for reproduction. Together, results indicate that low translation in different tissues helps direct distinct systemic adaptations and suggest that unknown endocrine signals mediate these responses. Furthermore, mTOR or translation inhibitory therapeutics that target specific tissues may achieve desired interventions to aging without loss of whole-body anabolism.

## INTRODUCTION

By nature, animals are driven by the need to feed and instinct to reproduce. When food is scarce or conditions are otherwise unfavorable for survival, anabolic activity is reduced and resource expenditure is redirected to preserve the soma (Liu and Qian, 2014). This helps ensure that animals survive long enough for conditions to improve and resume normal propagation. A robust and well-studied example of this is dietary restriction (DR), a low nutrient condition that extends lifespan and healthy tissue function, but with a cost to reproduction and growth (Shanley and Kirkwood, 2000).

The ability of cells to sense nutrients is central to the DR response. The mechanistic Target of Rapamycin (mTOR) is an ancient nutrient sensing serine/threonine kinase and subunit of the mTOR complex that controls processes including translation and autophagy (Kapahi et al., 2010). Low nutrient availability lessens activity through this pathway, reducing new protein synthesis and releasing suppression of cellular recycling. DR, low mTOR signaling, and genetically impaired translation each extend lifespan and stress resistance while negatively impacting growth and reproduction (Kapahi and Zid, 2004; Rogers and Kapahi, 2006; Kapahi and Vijg, 2009; Kapahi et al., 2010). This relationship is often considered in terms of life history trade-offs, a paradigm invoked in genetics of aging research in which reproductive success is “traded” for somatic maintenance (Maklakov and Chapman, 2019).

Some studies indicate that the trade-offs between reproduction and lifespan can be temporally uncoupled. In *D. melanogaster*, administration of the mTOR inhibitor Torin1 subsequent to mating does not decrease fecundity despite increasing lifespan (Mason et al., 2018). In *C. elegans*, animals heterozygous for translation initiation factor mutations can extend lifespan without strongly blunting fecundity (Cattie et al., 2016). Attenuation of the insulin/insulin-like growth factor-1 (IGF-1) pathway also exhibits trade-offs between reproduction and lifespan that may be partly or completely uncoupled. While not directly sensing nutrients in the same way as mTOR, reduced signaling through the insulin/IGF-1 pathway is important for increased lifespan from intermittent fasting (Honjoh et al., 2009) and reduced food sensing through taste receptors (Alcedo and Kenyon, 2004). Inhibition of insulin/IGF-1 signaling *after* animals reach adulthood maintains normal reproduction despite increasing lifespan (Dillin et al., 2002). In addition, a heterozygous mutant of this pathway in *D. melanogaster* exhibits robustly increased lifespan without a loss to reproduction (Yamamoto et al., 2021). Thus, the lifespan benefits of attenuating signaling through these food-responsive pathways are temporally separable from reproductive deficits or can otherwise be ameliorated by modifying the severity of attenuation through heterozygosity.

Little is known about the role of individual tissues in mediating trade-offs associated with low translation conditions. Lowering anabolic capacity in the reproductive system would be expected to have deleterious effects on gamete and embryo development. Less is known about the role of other tissues and the effects on lifespan. We investigated how major tissues control phenotypes associated with low translation to better understand how systemic adaptation is partitioned among cell-types. We focused on translation regulation downstream of mTOR through the cap-binding complex (CBC).

The CBC is activated by mTOR in response to nutrient abundance (Kapahi et al., 2010; Thoreen et al., 2012), supporting growth, reproduction, and replacement of degraded proteins. It comprises three subunits including the eukaryotic translation initiation factor (eIF-)4A helicase, the eIF-4E 5’ mRNA methylated cap-binding factor, and the eIF-4G scaffolding factor. Within the CBC, eIF-4G mediates both cap-dependent and cap-independent translation (Translational control of, 2000). Although present in multiple homologs in most systems studied, only a single copy is present in *C. elegans*, where it is called IFG-1 (Howard and Rogers, 2014). The protein level of IFG-1 is controlled by nutrient availability in *C. elegans* (Rogers et al., 2011). With respect to the trade-offs associated with DR and low nutrient signaling, attenuating *ifg-1* gene expression increases lifespan and stress-resistance (Hansen et al., 2007; Pan et al., 2007; Howard et al., 2016) and reduces growth and fertility (Curran and Ruvkun, 2007; Pan et al., 2007). Here, we find that lowering *ifg-1* gene expression in neurons, germline, or hypodermis increases lifespan. Conversely, lowering translation in body muscle or intestine has no positive effect and, when suppressed throughout development as well as adulthood, decreases lifespan. Surprisingly, lowering translation in muscle accelerates development and increased fecundity, not just uncoupling, but reversing trade-offs that result from systemic attenuation.

## RESULTS

### Reduced Translation Increases Lifespan and Resistance to Nutrient Deprivation in a Tissue-Dependent Manner

To investigate tissue-specific effects of reduced translation, we used previously constructed and characterized transgenic strains in which RNAi works primarily in a single tissue-type. Strains with functional RNAi only in muscle, intestine, or hypodermis were created from mutants of the Argonaut *rde-1* gene by restoring expression selectively using tissue-specific promoters. *C. elegans* strain WM118 rescues *rde-1* driven by the myosin heavy chain gene *myo-3* promoter for RNAi in body muscle (Cai et al., 2011), NR222 rescues *rde-1* driven by the *lin-26* promoter in hypodermis (Cai et al., 2011), while VP303 rescues *rde-1* driven by *nxh-2* promoter specific to intestine (Espelt et al., 2005). The MAH23 strain was used for germline-specific expression, which contains a mutation in *rrf-1(pk1417)* encoding RNA-dependent RNA polymerase that eliminates most somatic RNAi processing (Simmer et al., 2003). TU3335 overexpresses *sid-1*, a dsRNA channel, in neurons under the *unc-119* promoter and exhibits little or no RNAi phenotype penetrance for other tissues (Calixto et al., 2010).

**FIGURE 1 |:**
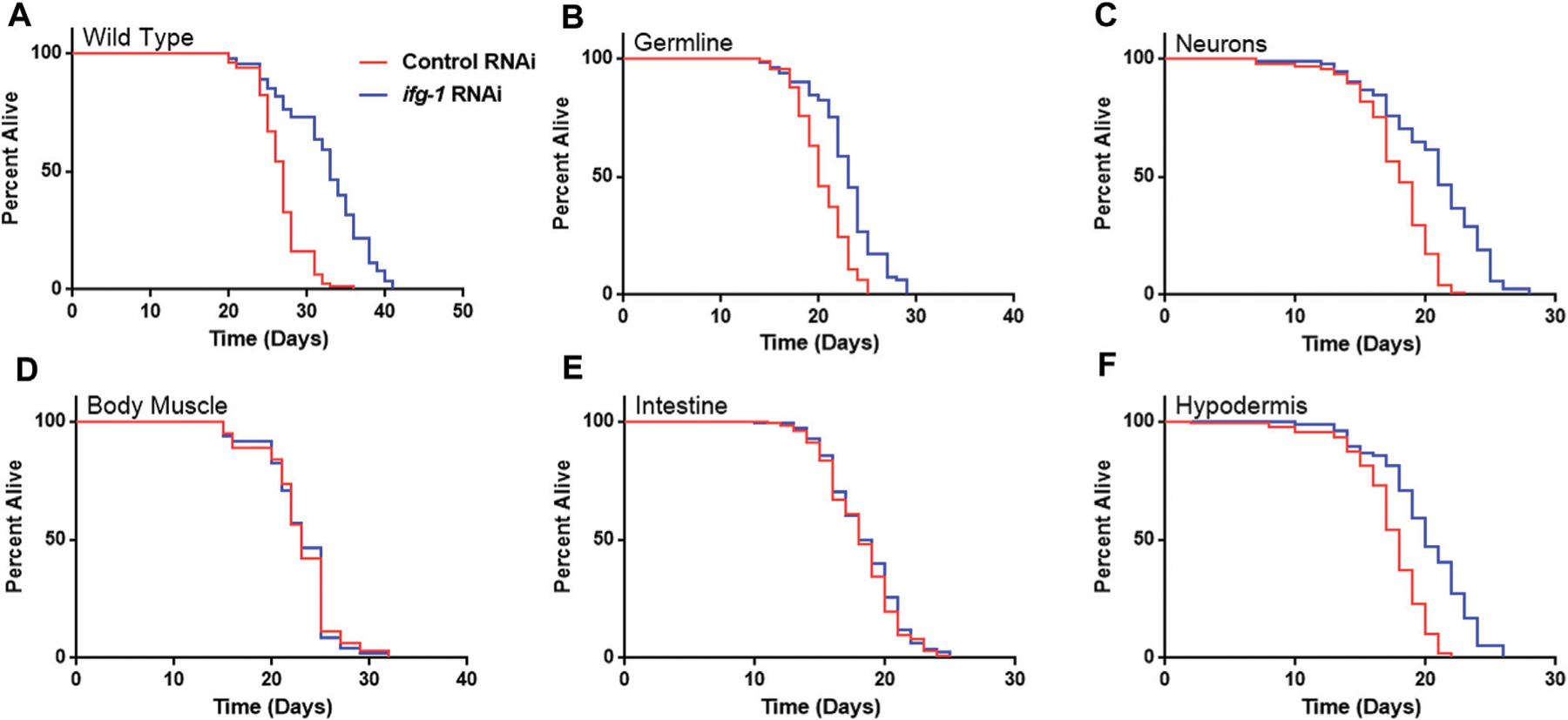
Reducing CBC-mediated translation selectively in germ cells, neurons or hypodermis extends lifespan. Bacteria expressing control or *ifg-1* dsRNA were fed to animals beginning at day 1 of adulthood in A-F. **(A)** Median lifespan in the wild-type N2 strain increased by 22.2% compared to control (*p* < 0.0001). **(B)** Median lifespan in the MAH23 germline-specific RNAi strain increased by 15.0% compared to control (*p* < 0.0001). **(C)** Median lifespan in the TU3335 neuronal-specific RNAi strain increased by 16.7% compared to control (*p* < 0.0001). **(D)** Median lifespan in the WM118 body muscle-specific RNAi strain was not significantly changed (*p* = 0.98). **(E)** Median lifespan in the VP303 intestine-specific RNAi was not significantly changed (*p* = 0.47). **(F)** Median lifespan in the NR222 hypodermis specific RNAi strain increased by 11.1% compared to control (*p* < 0.0001). Kaplan–Meier survival curves were compared using the Mantel–Cox log-rank test. See Supplementary Table S1 for additional details and replicates.

Strains were fed bacteria expressing dsRNA specific for *ifg-1* starting on the first day of adulthood. As observed in previous studies, lowering translation in wild-type N2 animals increased lifespan (Figure 1A), which was recapitulated to varying extents when lowered selectively in the germline, neurons, or hypodermis (Figure 1,B,C,F; Supplementary Table S1). In each case, the extent of lifespan increase was less than that observed for systemic translation repression in wild-type animals, ranging from just over a third of the wild-type in hypodermal suppression to almost two thirds in neuronal *ifg-1* knock-down. Polysome profiling confirmed translation was reduced in intestine- and body muscle-specific RNAi strains targeting *ifg-1* (Supplementary Figure S1). Results show that only lowering CBC-mediated translation in particular tissues increases lifespan.

In light of the robust lifespan extension from lowering translation in reproductive tissue and the role the germline is known to play in *C. elegans* longevity generally (Hsin and Kenyon, 1999), we tested whether the absence of a developed germline eliminates lifespan extension in this model. For this, a temperature-sensitive germline-deficient *glp-4(bn2)* mutant was used, which was shown previously to contain only 12 germ cells arrested at the prophase stage of mitosis when development is carried out at 25°C (Beanan and Strome, 1992). Lowering *ifg-1* in this strain increased median lifespan by 30% (Supplementary Figure S2; Supplementary Table S2). Thus, inhibiting proliferation in the germline is not required for increased lifespan under low translation conditions.

**FIGURE 2 |:**
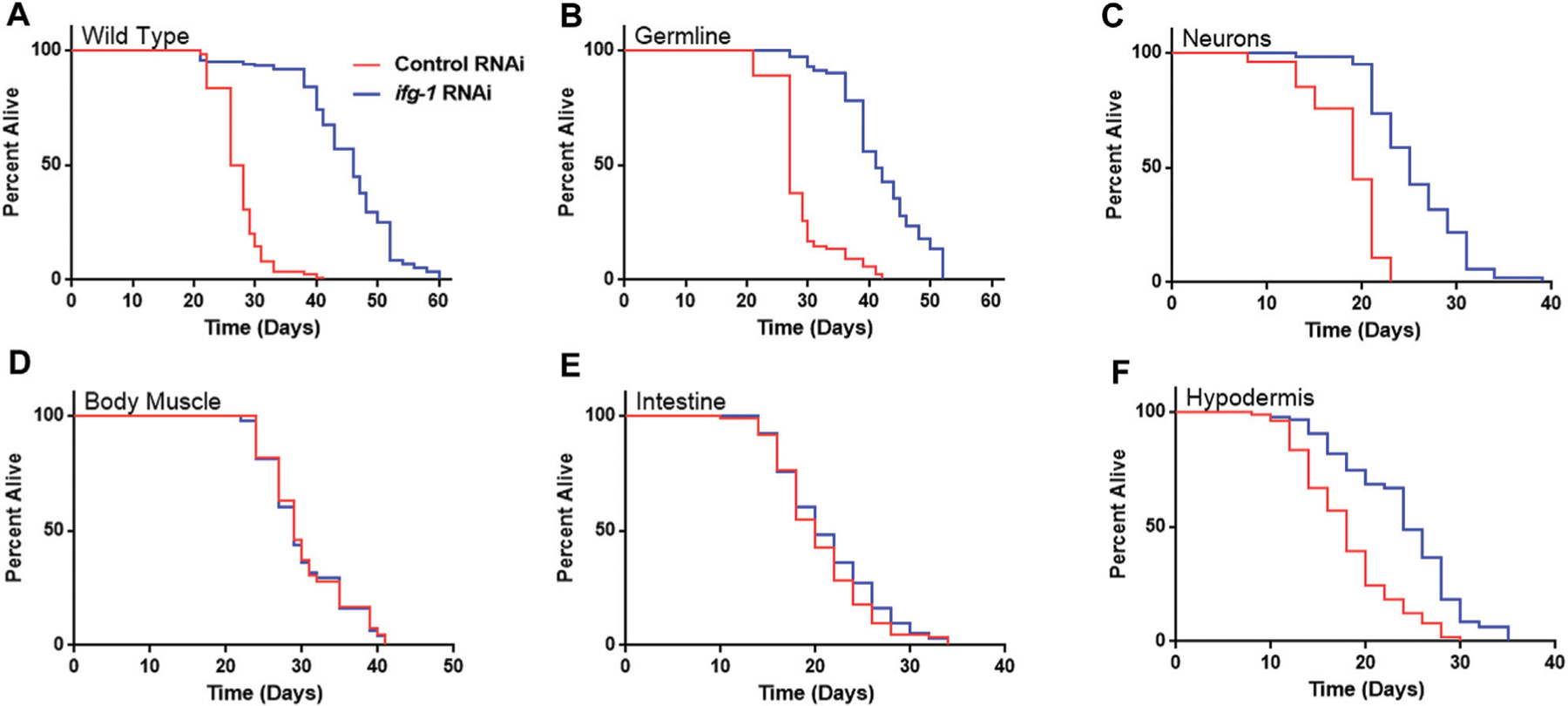
Survival under nutrient deprivation is increased in a tissue-specific manner when *ifg-1* is reduced in adulthood. Worms were fed bacteria expressing control or *ifg-1* dsRNA for 2 days beginning at adulthood. Nutrient deprivation was initiated by complete removal from food. **(A)** Median lifespan in the N2 wild type strain increased by 76.9% compared to control (*p* < 0.0001). **(B)** Median lifespan in the MAH23 germline-specific RNAi strain increased by 51.9% compared to control (*p* < 0.0001). **(C)** Median lifespan in the TU3335 neuron-specific RNAi strain increased by 31.6% compared to control (*p* < 0.0001). **(D)** Median lifespan in the WM118 muscle-specific RNAi strain was not significantly changed (*p* = 0.91). **(E)** Median lifespan in the VP303 intestine-specific RNAi strain was not significantly changed (*p* = 0.28). **(F)** Median lifespan in the NR222 hypodermis-specific RNAi strain increased by 33.3% compared to control (*p* < 0.0001). Kaplan–Meier survival curves were compared using the Mantel–Cox log-rank test. See Supplementary Table S3 for additional details and replicates.

Given the relationship between translation and nutrient signaling, we tested the effect of lowering tissue specific translation upon withdrawal of food and found that, again, germline, hypodermis and neurons controlled increased survival (Figure 2; Supplementary Table S3). Lowering translation in neurons or germ cells had nearly equal effect that was about half the increase observed in wild-type N2. Results suggest that the same mechanism(s) underlie control of lifespan and resistance to starvation when translation is attenuated in these tissues. As with lifespan, survival without food was enhanced fractionally in tissue-specific RNAi strains compared to the N2 wild-type strain. Lifespan and starvation survival effects from low translation in these tissues may be cumulative or represent differences in RNAi efficacy between tissue-specific RNAi strains and wild-type animals for the same tissue.

To compare effects of tissue-specific RNAi with knockdown in the same tissue of the wild-type strain, we chose to focus on body muscle because it was one of two tissues that, when selectively targeted for translation inhibition during adulthood, showed no effect on lifespan. For this, we employed a movement assay targeting *unc-22*, a twitchin gene that regulates actomyosin contraction and relaxation (Moerman and Baillie, 1979). In a comparison among tissue-specific RNAi strains, results showed motility was most affected in the body muscle-specific RNAi and N2 wild-type animals, with no discernable effect in germline, intestine, and hypodermis-specific RNAi strains (Supplementary Figure S3A). Another test of RNAi efficacy showed similar knockdown of GFP driven by the myosin heavy chain *myo-3* promoter in wild-type and body muscle specific RNAi strains (65.63 ± 0.98% and 71.72 ± 1.02%, respectively; Supplementary Figure S3B).

**FIGURE 3 |:**
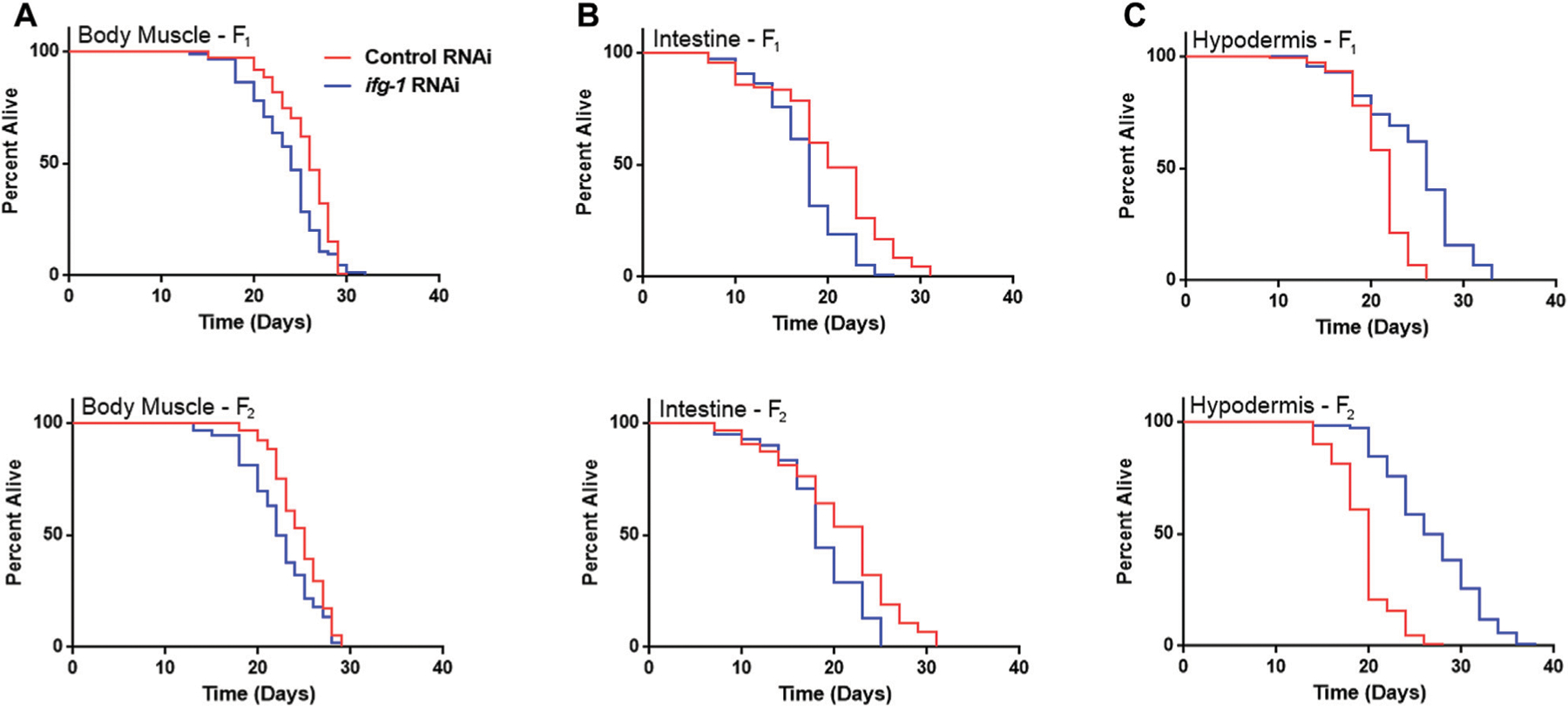
Developmental and multi-generational knockdown of *ifg-1* in body muscle or intestine decreases lifespan. Low translation in F1 (top panel) and F2 (bottom panel) generations by continuous exposure to bacteria bearing dsRNA of *ifg-1* or control was carried out in tissue-specific RNAi strains. **(A)** Median lifespan in the WM118 body muscle-specific RNAi strain decreased by 7.7% in the F1 generation (*p* = 0.0013) and by 8.0% in the F2 generation (*p* < 0.0001). **(B)** Median lifespan in the VP303 intestine-specific RNAi strain decreased by 10.0% in the F1 generation (*p* < 0.0001) and by 21.7% in the F2 generation (*p* = 0.0002). **(C)** Median lifespan in the NR222 hypodermis-specific RNAi strain increased by 18.2% in the F1 generation (*p* < 0.0001) and by 35.0% for the F2 generation (*p* < 0.0001). Kaplan–Meier survival curves were compared using the Mantel–Cox log-rank test. See Supplementary Table S4 for additional details and replicates.

Developmental and multigenerational suppression of translation through *ifg-1* was used to see if increasing exposure has an effect on lifespan in body muscle- and intestine-specific RNAi strains. Late-larval staged animals were placed on bacteria expressing dsRNA targeting *ifg-1* and allowed to reproduce. F1 and F2 progeny were raised throughout development and adulthood under the same conditions. Both strains showed decreased lifespan for F1 and F2 generations (Figures 3A,B, respectively, Supplementary Table S4). Conversely, using this approach in the hypodermis-specific RNAi strain further increased lifespan compared with adult-only exposure (Figure 3C; Supplementary Table S4). Wild-type N2 could not be included for comparison due to sterility caused by lowering translation. Together with differences observed in Figure 1, results demonstrate that lifespan changes under low translation conditions are tissue-dependent and can have opposing effects on lifespan.

### Trade-Offs Between Lifespan and Both Reproduction and Development are Reversed when Translation is Selectively Reduced in Body Muscle

**FIGURE 4 |:**
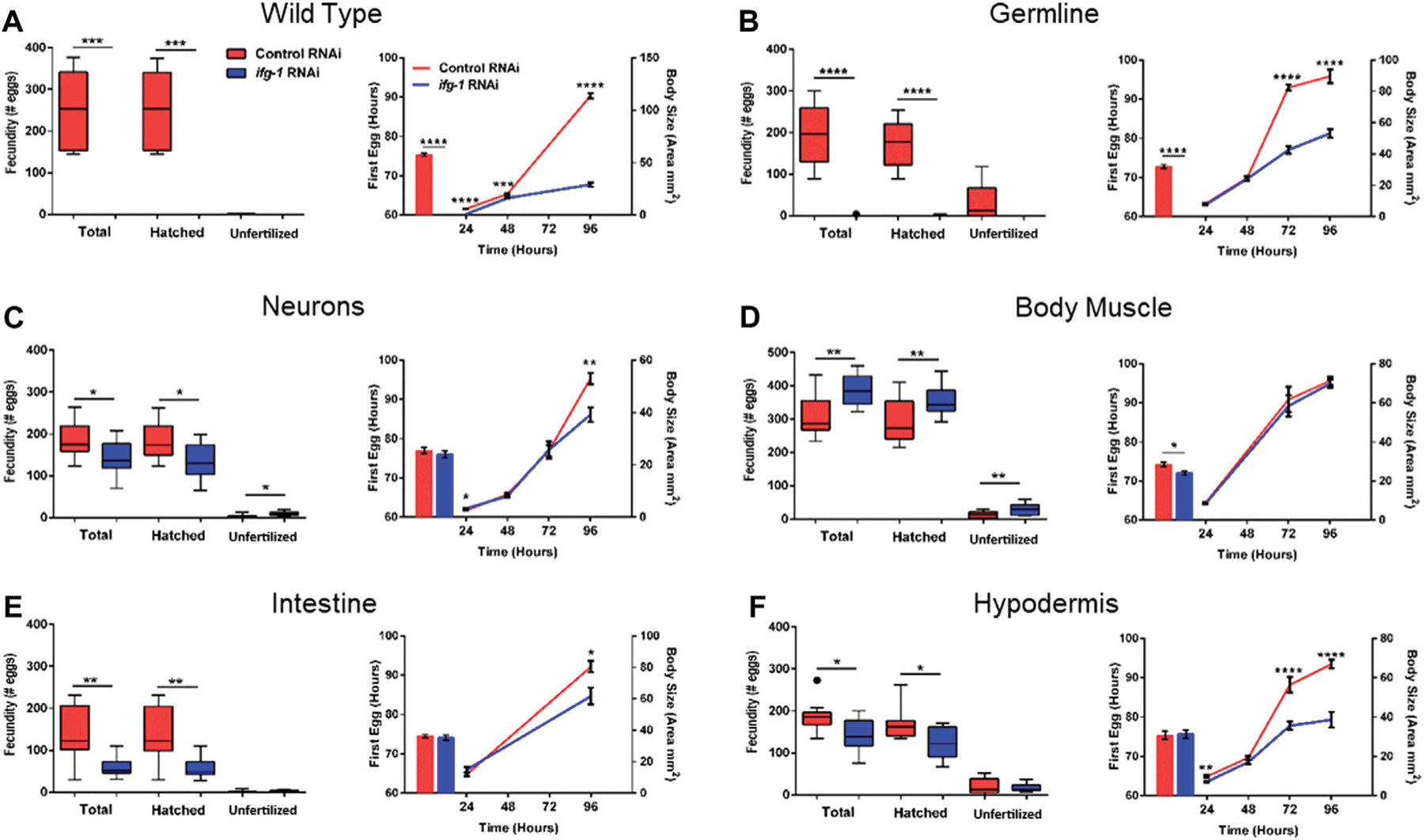
Reduced translation in muscle has opposing effects on reproduction and growth compared with reduced translation in other tissues. Gravid adults were placed on bacteria expressing control or *ifg-1* dsRNA and allowed to lay eggs for 1 h. Adults were removed and larvae monitored for growth and fecundity. Reproduction was measured separately for hatched progeny and unfertilized eggs (left panel). Sexual maturation rate was measured from the time an egg was laid through development to becoming an egg-laying adult (right panel). Growth rate was determined by monitoring body size for 96 h after being laid (right panel). **(A)** Knockdown of *ifg-1* in N2 wild type animals significantly diminished fecundity, development, and growth. **(B)** Germline knockdown of *ifg-1* also significantly decreased fecundity, development, and growth. **(C)** Knockdown of *ifg-1* in the neuron-specific RNAi strain significantly decreased total and hatched offspring, had no effect on development and significantly reduced growth. **(D)** Knockdown of *ifg-1* in the body muscle-specific strain significantly increased fecundity, reduced the time to reproductive adulthood, and had no effect on growth. **(E)** Intestinal knockdown of *ifg-1* significantly decreased fecundity and growth with no significant change in development. **(F)** Hypodermal knockdown of *ifg-1* significantly decreased total fecundity and growth with no significant change in the rate of development. 9–16 animals per condition were assessed for reproduction and growth at each time point. 8–19 animals were assessed for development. All assays were replicated with similar results. Error bars on bar and line graphs (right panels) represent standard error of the mean. Significance in Tukey box plots (left panels) as well as bar and line graphs was calculated using unpaired t-test with Welch’s Correction (*p < 0.05, **p < 0.01, ***p < 0.001, ****p < 0.0001). Data points outside the whiskers of the box plot are shown. Data were pooled from two experiments.

To test whether trade-offs between lifespan and growth-related processes were also tissue-dependent, we measured fecundity and the rate of development under conditions of low translation in select tissues. Development rate was determined by changes in size with age as well as the time it took for animals to become egg-laying adults (Figure 4; Supplementary Tables S5, S6). Wild type N2 animals grown in the presence of *ifg-1* RNAi were unable to reproduce and showed delayed growth (Figure 4A, left and right panels, respectively). Failure to reproduce and a less severe deficit in growth was observed under germline-specific translation reduction (Figure 4B). Low translation in hypodermis also delayed growth (Figure 4F), whereas minor growth delays were observed in neuron- and intestine-specific RNAi strains (Figures 4C,E, respectively). Interestingly, low translation in the intestine-specific strain resulted in a pronounced reproductive deficit, which is positively correlated with decreased lifespan observed in Figure 3. Surprisingly, fecundity and rate of development to reproductive adulthood increased significantly when translation was lowered selectively in body muscle (Figure 4D). Thus, trade-offs between reproduction and lifespan are uncoupled for translation attenuation in intestine and reversed for low translation in body muscle.

Sperm is the limiting gamete in *C. elegans* hermaphroditic reproduction and is generated late in development (Ward and Carrel, 1979). We performed sperm counts in young adults immediately following passage of the first egg through the spermatheca to determine whether this could help explain the changes in fecundity. In agreement with no progeny observed in wild-type N2 raised on *ifg-1* RNAi in Figure 4, no sperm were observed under this condition (Supplementary Figure S3). A small number of sperm were observed in the germline-specific RNAi strain raised in the presence of *ifg-1* RNAi, which is also in agreement with a small number of progeny by some animals before egg-laying ceased under this condition in Figure 4B. Finally, body muscle-specific *ifg-1* knockdown significantly increased sperm count (Supplementary Figure S4). Results show that changes in the number of sperm reflect changes in fecundity when translation is selectively attenuated in germline or body muscle.

### Low mTORC1 or Cap-Binding Complex-Mediated Translation in Body Muscle Increases Reproduction

**FIGURE 5 |:**
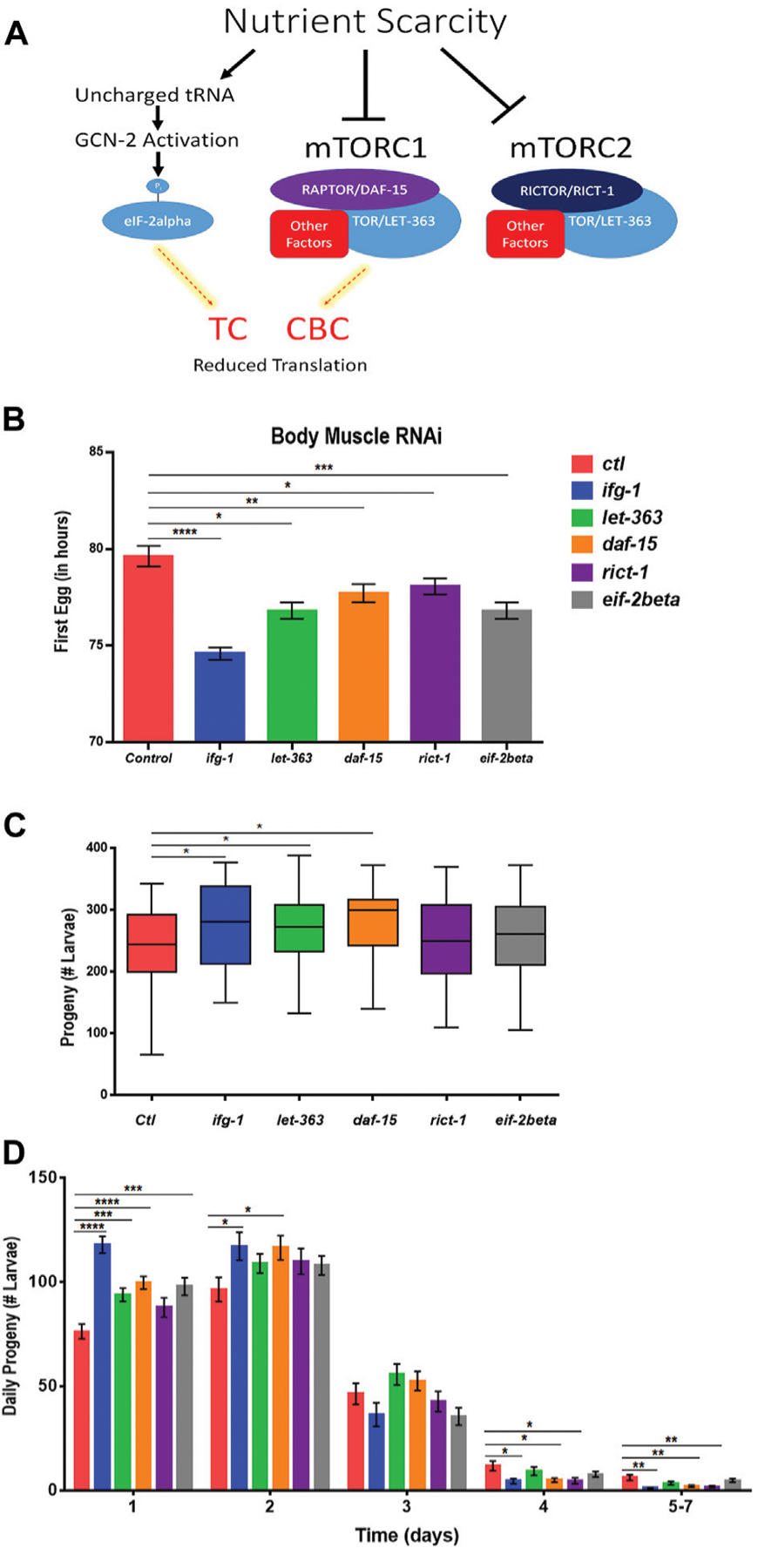
Restricting mTORC1 or translation downstream of mTORC1 selectively in body muscle increases fecundity. **(A)** Schematic showing the two major arms of translation initiation influence by nutrient limitation. The CBC is driven by signals from mTORC1, while the TC is independent of mTOR. Thin dashed red lines highlighted in yellow indicate reduced activity. **(B–D)** Larvae for the WM118 body muscle-specific RNAi strain were raised on bacteria expressing dsRNA for genes targeting subunits of mTORC1, mTORC2, and the TC. The average time to reproductive adulthood is shown in **(B)**, whereas **(C)** shows the average number of viable progeny and **(D)** shows the average daily number of progeny for each condition. Error bars in **(B)** and **(D)** represent the standard error of the mean. Significance in bar graphs **(B,D)** and Tukey box plots **(C)** was calculated using unpaired t-test with Welch’s Correction (*p < 0.05, **p < 0.01, ***p < 0.001, ****p < 0.0001). Data were pooled from three experiments (Supplementary Tables S7, S8).

Some translation regulators are not regulated by mTOR. Conversely, some mTOR functions are independent of translation. Thus, we sought to understand whether the effects of body muscle translation on reproduction were specific to IFG-1 and the CBC. Translation initiation is regulated by the mTOR-driven CBC and by the mTOR-independent ternary complex (TC). The ternary complex comprises eIF-2, GTP, and methionyl-tRNA required to begin translation (Rollins et al., 2017). In response to various forms of stress and nutrient scarcity, the alpha subunit of eIF2 is phosphorylated, preventing GDP-to-GTP recharging and blocking new translation. A schematic representation of the influence of nutrient limitation on mTOR, the CBC, and the TC is shown in Figure 5A. The mTOR pathway is directed by two multi-subunit complexes, the rapamycin-sensitive mTORC1 and rapamycin-insensitive mTORC2 (Loewith et al., 2002). Each has different subunits as well as some in common, with the latter including the core mTOR subunit, called LET-363 in *C. elegans*. mTORC1 drives protein synthesis and blocks autophagy, although both complexes are important for growth and development (Blackwell et al., 2019). In *C. elegans*, the mTORC1-specific Raptor subunit is known as DAF-15 and mTORC2-specific Rictor subunit as RICT-1. Genes encoding mTORC and TC subunits were knocked down throughout development in the body muscle-specific RNAi strain. Attenuating activity of either mTOR complex or the TC by targeting genes encoding subunits for these complexes reduced the time to becoming an egg-laying adult (Figure 5B). However, reproduction was only increased when expressions of genes encoding the mTORC1 subunits LET-363 and DAF-15 were targeted with RNAi (Figure 5C). Consistent with accelerated onset of reproduction under low mTOR/translation in body muscle, reproduction was also completed earlier under these conditions (Figure 5D). Together, results indicate that reproductive development and fecundity are negatively influenced by translation downstream of the mTORC1 branch of the mTOR pathway in body muscle.

### Low mTORC1 in Body Muscle or Germline Have Opposite Effects on Motility During Development

**FIGURE 6 |:**
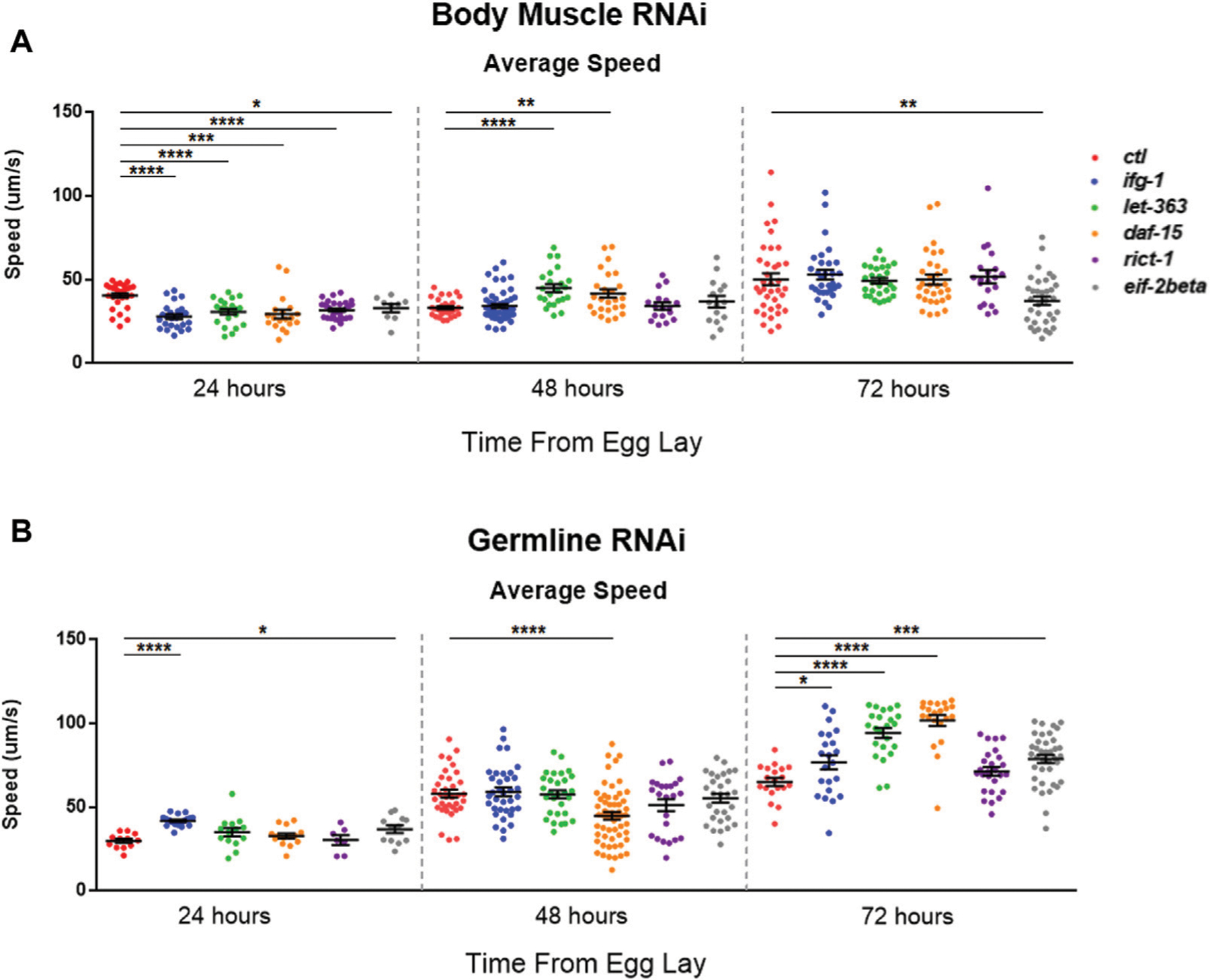
Restricting mTOR in the germline increases motility while restriction in body muscle decreases motility.Average speed was measured on animals fed bacteria expressing dsRNA throughout development for the genes shown. **(A)** Motility in the WM118 body muscle-specific RNAi strain at 24-, 48-, and 72-h. **(B)** Same as in **(A)**, but for motility in the MAH23 germline-specific RNAi strain. In each experiment, >75 worms were tracked for 45s. Scatter plots display bars representing mean and standard error of the mean for all conditions. Statistical comparisons were done using two-tailed unpaired t-tests with Welch’s Correction (*p < 0.05, **p < 0.01, ***p < 0.001, *****p < 0.001). See Supplementary Table S9 for more information.

mTOR and translation are stimulated subsequent to resistance and endurance exercise (Bodine et al., 2001; Goodman et al., 2011), suggesting that mTOR activation may behave as a proxy for muscle activity. We wondered whether the inverse scenario, one in which the optimal potential for mTOR activity was blunted in *C. elegans* body muscle during development, would lead to reduced motility. Average speed was assessed during development at 24-h (early larval), 48-h (mid-late larval), and 72-h (young adult) after eggs were laid on bacteria expressing dsRNA targeting mTOR/translation-related genes. Average speed was blunted at 24-h in animals subjected to low mTOR/translation in body muscle (Figure 6A). When considered with observations from reproductive assays, results indicate that low mTORC1 activity in body muscle controls the inverse relationship observed between fecundity and motility. We wondered whether low germline mTOR/translation also produced an inverse relationship between reproduction and motility. Low mTORC1, CBC, or TC activity significantly increased motility by young adulthood in the germline tissue-specific RNAi strain (Figure 6B).

## DISCUSSION

### Organismal Responses to Low Translation Are Partitioned Among Tissues

Genetically restricting mTOR or translation results in the same inverse relationship between reproduction and lifespan observed under dietary restriction (Kapahi et al., 2010). Previous evidence shows that this relationship can be temporally uncoupled by lowering mTOR or translation after growth and reproductive development are complete (Dillin et al., 2002; Mason et al., 2018). This study addresses the roles of major tissues in mediating effects of low translation. We find that these phenotypes can also be uncoupled according to tissue type and, in the case of body muscle, reversed.

**FIGURE 7 |:**
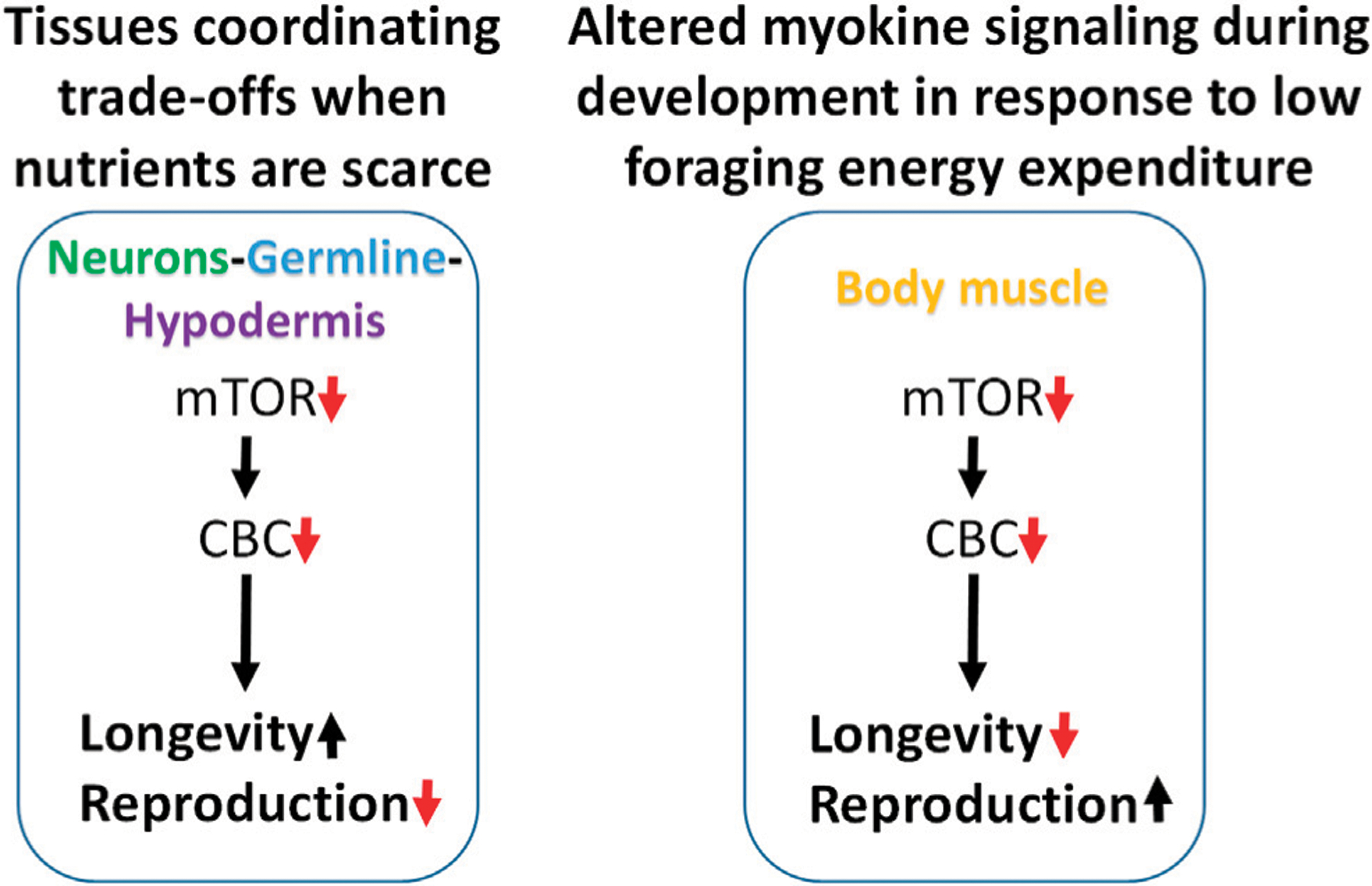
Modeling trade-offs between nutrient sensing, lifespan and reproduction. Low translation through the CBC downstream of mTOR increases lifespan and reduces fecundity when carried out selectively in neurons, germline, or hypodermis, suggesting that these tissues may be responsible for coordinating somatic protection under low nutrient conditions (left). Conversely, low translation in body muscle can shorten lifespan and increase reproduction under normal feeding conditions. Given the positive connection between muscle use and translation, and that less energy is expended on foraging when nutrients are abundant, low translation in body muscle may be a signal that conditions are highly conducive for maximizing reproductive output (right).

Attenuating translation in neurons, hypodermis, or germline is sufficient to increase lifespan and reduce fecundity. We propose a model in which these tissues enhance somatic resilience that increases lifespan in response to low translation conditions associated with nutrient scarcity (Figure 7). Evidence of tissue cross-talk that may be necessary for increased lifespan in this model is consistent with changes in motility observed when translation is reduced in tissue other than body muscle, such as the germline. Compared with systemic translation suppression, fractional effects on lifespan from low translation in a single tissue are consistent with signal amplification expected when multiple tissues are involved. In this model, the smaller, negative effects on lifespan from low translation in body muscle or intestine are overcome by the much stronger positive effects coming from the germline, neurons, and hypodermis.

In the second part of this model, the response to low translation only in body muscle reflects a different kind of environmental condition. We showed that low mTORC1 or translation in body muscle decreases motility and increases reproduction. Motility involves muscle contractions such as those required for food-seeking. In behavioral ecology, optimal foraging theory predicts that reproductive fitness is maximized when a species adopts a foraging strategy involving the greatest gains for the least work. That way, extra energy can go toward the expense involved in reproduction (Werner and Hall, 1974; Raubenheimer and Simpson, 2018; Abrahms et al., 2021). Animal and human studies show that anabolic activity increases in skeletal muscle following exercise (Bodine et al., 2001; Wilkinson et al., 2008; Goodman et al., 2011). If persistent low translation in body muscle is indicative of inactivity, it suggests that changes in signaling from this tissue may be responsible for increasing the rate of development and reproductive output. When coupled with an ample food supply, low muscle translation may be a cue indicating that foraging energy use is low and that conditions are optimal for reproduction (Figure 7).

There is precedent for muscle directing certain germline characteristics. During development, proper growth of the gonad distal tip in *C. elegans* requires pro-growth signaling from the muscle by a secreted metalloprotease called GON-1 (Blelloch and Kimble, 1999). In other systems, muscle has become increasingly appreciated for its role as a source of endocrine signals. Small peptides released by muscle during contraction are called myokines (Pedersen et al., 2003) and have hormone-like effects with receptors in diverse tissues. Although a relatively small number of them are formally characterized, evidence from mammalian studies suggest that there may be hundreds (Bortoluzzi et al., 2005; Yoon et al., 2009; Henningsen et al., 2010). Ones already identified have key roles in metabolic health and include interactions with adipose tissue, liver, pancreas, cardiovascular system, brain, bone, and skin. Many myokines are dependent on contraction for their production and release, providing a possible mechanism for the link between sedentary behavior and age-related chronic diseases (Pedersen and Febbraio, 2012).

Alternatively, inter-tissue communication may not be responsible for the phenotypes observed. For example, if neurons, hypodermis, and germline were lifespan-limiting, low translation may improve health by enhancing maintenance of only those tissues. However, the germline is only needed for reproduction, not viability. While neurons are essential, aging appears to effect this tissue more slowly than muscle (Herndon et al., 2002), another essential tissue but one for which low translation does not increase lifespan. Yet another possibility is that there is widespread RNAi “leakiness” in the strains used such that translation is still attenuated systemically, albeit at a lower level than in wild-type animals. However, this is not supported by RNAi efficacy and specificity tests carried out here or in other studies. One exception is the germline-specific RNAi strain MAH23, which was shown to have some activity in the intestine and hypodermis (Kumsta and Hansen, 2012). However, lowering translation in this strain or the intestine-specific RNAi strain VP303 had opposite effects on lifespan. Additionally, the extended lifespan in hypodermis was not as great as that shown for germline-specific suppression of translation, indicating that hypodermis-specific effects observed in the NR222 strain cannot explain the more extreme phenotypes observed in MAH23. It is also well known that conditions that give rise to germ cell loss, like the low translation conditions used in the present study, increase lifespan (discussed in the next section). Finally, neither cell autonomous effects nor imperfect tissue specificity of RNAi can explain how lowering mTOR or translation in muscle increases fecundity.

### The Role of the Germline in Aging

Reproductive organs regulate lifespan in diverse eukaryotic systems (Leopold et al., 1959; Arantes-Oliveira et al., 2002; Flatt et al., 2008), including mammals (Mason et al., 2009; Min et al., 2012). In *C. elegans*, ablating germ cell precursors increases lifespan (Hsin and Kenyon, 1999). Hormone signaling from the somatic gonad leads to intestinal activation of DAF-16, the FOXO-family transcription factor responsible for increased lifespan when insulin-like signaling is attenuated (Lin et al., 2001; Libina et al., 2003). Increased lifespan through loss of germ cells also requires signals originating from the somatic gonad during development to the steroid hormone receptor DAF-12 (Gerisch et al., 2001; Yamawaki et al., 2010) as well as expression of miR-71 in neurons (Boulias and Horvitz, 2012). Thus, the effect on longevity through the *C. elegans* reproductive system is a coordinated effort that requires tissue cross-talk. While lifespan extension by restricting translation systemically does not require DAF-16, it is necessary to maximize the extension when *ifg-1* gene expression is restricted systemically (Rogers et al., 2011). It is interesting to note that unlike increased lifespan from hormone signaling changes due to germ cell loss, low translation in germline increases lifespan *after* development is complete (Figure 1). Furthermore, we find that altered signaling from an intact germline is not required for low translation conditions to increase lifespan. Additional studies are required to determine how closely lifespan changes via the germline and these pathways are related.

### The Connection Between Reproduction and Translation in Hypodermis and Intestine

In this study, germline or systemic RNAi-mediated knockdown of eIF-4G/*ifg-1* shut down sperm production during development. A separate study showed that oogenesis is inhibited when *ifg-1* expression is reduced (Contreras et al., 2008). Negative effects on germline processes when anabolic activity is reduced is not surprising. However, we also observed small but significant decreases in fecundity when translation is lowered selectively in intestinal or hypodermal cells.

Disrupting translation in intestine could be detrimental to reproduction for the simple fact that the gut processes nutrients for all other tissues. Yolk synthesis required to nourish the germline is synthesized in the intestine (Kimble and Sharrock, 1983), which is also the primary fat storage tissue in *C. elegans*. Mobilization of intestinal fat stores to nourish the germline is controlled by a microRNA circuit in the hypodermis (Dowen et al., 2016). Interestingly, this involves mTORC2-specific signaling in the intestine. Disrupting core intestinal cellular activity, including translation, induces behavioral changes that suggest a neuroendocrine axis of control (Melo and Ruvkun, 2012). In other animal systems, including humans, adipose tissue and its endocrine functions positively influence reproduction (Friedman, 1998; Chehab, 2014; Bornelöv et al., 2018). Since low translation through eIF4G/IFG-1 leads to intestinal atrophy (Long et al., 2002), it is likely that both yolk synthesis or fat stores are diminished and likely to decrease reproductive potential.

While low translation in hypodermis results in lifespan and fecundity changes in line with trade-offs associated with Disposable Soma theory (Kirkwood, 1977), low translation in the intestine does not. This is not to say that changes in the intestine associated with reduced anabolism are incapable of producing trade-offs associated with the theory. For example, inhibiting vitellogenin genes expressed in the intestine that encode yolk proteins increases lifespan (Murphy et al., 2003). However, it does suggest that the net effect on lifespan from lowering intestinal translation is not driven by its influence on fecundity. Additionally, the observation that low translation in hypodermis or germline both reduce fecundity but have different effects on lifespan argues against passive changes in energy redistribution as drivers of differential effects and argues for coordinated responses based on tissue function.

### Motility Under Conditions of Low Nutrient Signaling

The motility response to low translation in body muscle is particularly interesting with respect to behavior intended to support survival during times of nutrient scarcity. When food is unavailable, protein synthesis is attenuated and animals enter a catabolic state in which cellular constituents are recycled to supply energy and raw materials necessary to keep tissues viable. However, lack of food also stimulates changes in foraging behavior. For many animals, food seeking requires keeping muscle tissue highly functional despite nutrient scarcity. In experiments demonstrating behavioral plasticity in *C. elegans*, removing food promoted motility, whereas making food available resulted in slowed movement (Sawin et al., 2000). Such behaviors are mediated by neurotransmitters that also influence behavior in more complex systems.

In a generalized context, nutrient deficiency and the costly process of translation are at odds. Indeed, inhibiting the nutrient responsive mTORC1 with the drug rapamycin can block anabolic effects from contractions associated with mechanical adaptive overload in muscle tissue (Bodine et al., 2001; Goodman et al., 2011). However, while it blocks hypertrophy, rapamycin does not promote atrophy (Bodine et al., 2001). When low mTOR signaling is coupled/driven by nutrient scarcity, it increases mitochondrial respiration in skeletal muscle and mitigates normal respiratory decline observed with age in mice (Hempenstall et al., 2012). Evidence also indicates that muscle is shielded somewhat from low nutrient signaling conditions. Whereas high dosage of the TOR inhibitory drug rapamycin had a negative effect on translation in muscle, low doses (<10 ug/kg body weight) actually stimulated anabolic processes in this tissue (Lee et al., 2014). Authors of that study suggested that this result may be due to changes in calcium signaling and storage that take place below the threshold for TOR inactivation. Another possible contributing factor supported by findings made here is that other tissues help coordinate responses in muscle. Whereas lowering mTORC1-related genes in body muscle had a negative effect on motility, knock-down of the same genes in the reproductive system throughout development increased average speed by early adulthood. Given the number of studies showing positive effects on muscle with age and age-related disease in animals administered rapamycin, it is worth considering whether some effects in muscle may be due to cross-talk with other tissues. We propose that low translation in body muscle is read as a signal of low physical activity, promoting energy storage and signaling optimal conditions for reproduction.

Future studies are needed to show how anabolic function downstream of mTORC1 initiates different cross-talk signals and outcomes from different tissues.

## MATERIALS AND METHODS

### Nematode Culture and Strains

*C. elegans* strains were cultured and maintained with standard procedures as described in (Brenner, 1974) unless otherwise specified. The N2 Bristol strain was used as the reference wild type. Strains acquired from the Caenorhabditis Genetics Center are as follows: SS104 *glp-4(bn2)*, MAH23 *rrf-1(pk1417)*, VP303 *rde-1(ne219)*; kbIs7[*nhx-2*p∷*rde-1* + *rol-6(su1006)*], NR222 *rde-1(ne219)*; kbIs9[*lin-26p*∷nls∷GFP, *lin-26p*∷*rde-1* + *rol-6(su1006)*], WM118 *rde-1(ne300)*; neIs9[*myo-3*p∷HA∷*rde-1* + pRF4(*rol-6(su1006)*)], TU3335 *lin-15B(n744)*; uIs57 [*unc-119*p∷YFP + *unc-119*p∷*sid-1* + *mec-6*p∷*mec-6*]. ANR101 rogEx101 [*myo-3p*∷GFP] and ANR103 rogEx103 [*myo-3*p∷GFP]; *rde-1(ne300)*; nels[*myo-3*p:HA∷*rde-1* + pRF4(*rol-6(su1006)*)] were generated in wild-type N2 and WM118 using microinjection to create extrachromosomal arrays according to the protocol in Wormbook.org.

### RNA Interference Experiments

RNAi knockdown experiments treatments were performed as previously described (Kamath, 2003). Efficacy of RNAi knockdown was verified by observing growth and reproductive deficits in wild-type N2 larvae for each gene target. Efficacy of RNAi in body muscle was tested by targeting *unc-22* via RNAi and measuring the frequency of abnormal motility in adults after 1 week. It was also tested in wild-type N2 and WM118 animals expressing GFP in body muscle by measuring the difference in fluorescence after 1 week of RNAi targeting GFP or control (L4440) vector. RNAi bacteria strains included empty vector L4440, GFP: L4440 (11335) (Addgene, Cambridge, MA, United States), *eif-2beta* (K04G2.1), *ifg-1* (M110.4), *let-363* (B0261.2), *unc-22* (ZK617.1) from the Ahringer library (Source BioScience, Nottingham, United Kingdom) and *rict-1* (F29C12.3) from the Vidal library (Source Bioscience). *daf-15* (C10C5.6) (Zhu et al., 2015) was a generous gift from the Han Lab (University of Colorado, Boulder).

### Analysis of Lifespan and Survival Upon Food Withdrawal

Nematodes were obtained by bleaching gravid adults and allowing them to hatch overnight in S basal (0.1 M NaCl, 5.74 mM K2HPO4, 44.09 mM KH2PO4, 1 ml cholesterol (5 mg/ml in ethanol), in H2O to 1 L). Synchronized L1 larvae were placed on NGM plates spotted with OP50. Upon reaching adulthood, worms were transferred to NGM “RNAi” plates containing 1 mM isopropylthio-β-galactoside (IPTG), 25 mg/ml carbenicillin, and 50 μg/ml 5-fluoro-2-deoxyuridine (FUdR) to inhibit egg production. In food withdrawal experiments, day 1 adults were placed on *ifg-1* RNAi media for 2 days. They were then washed in phosphate buffer 6 times, allowing them to settle via sedimentation between each wash. They were then transferred to fresh RNAi media without peptone or bacteria. For multi-generational lifespan analysis, animals were synchronized by a 2-h timed egg lay, and eggs were permitted to grow until adulthood. F1 lifespan was determined by timed egg lay on RNAi in the absence of FUdR. Upon reaching adult day 1, F1 worms were shifted to fresh RNAi plates containing FUdR. F2 worms were obtained from a timed egg lay on RNAi using gravid adults maintained from the first timed egg lay on RNAi. F2 worms were transferred to fresh RNAi treated with FUdR on day 1 of adulthood. Germline-deficient SS104 *glp-4(bn2)* worms were cultured and maintained on OP50 at 15°C. Late L4 worms were placed onto OP50 and shifted to 25°C for an overnight timed egg lay prior to removal from the plate. Progeny were maintained at 25°C until adulthood to arrest germline development. Day 1 adults were placed on control or *ifg-1* RNAi plates without FUdR and maintained at 25°C for the duration of the lifespan. In all lifespan experiments, worms were scored for live versus dead daily by gently tapping worms with a platinum wire. Worms that failed to respond to several taps were scored as dead and removed from the plate. Worms were censored if they died because of vulval rupture or desiccation from moving off the culture plate. Lifespan experiments were performed at 20°C except for germline temperature-sensitive *glp-4(bn2)* mutants.

### Sexual Development and Fecundity Analysis

Sexual development was assessed by measuring the time between when an egg was laid until it developed into an egg-laying adult. For this, young gravid adults were placed on bacteria expressing dsRNA for 2-h to obtain a similarly staged cohort. 48 h later, individual progeny were moved to fresh media with one animal per plate to monitor reproduction. The onset of reproduction was determined by monitoring late larval stage worms every hour until the first egg was expelled from the body (first egg lay). Total hatched and unfertilized eggs were counted for 7 days. To determine daily fecundity rates, a 1-h timed egg lay was performed to synchronize embryos. However, we observed that the WM118 body muscle-specific RNAi strain exhibited defective egg laying in which developing embryos in the early gastrulation stage were sometimes mixed with late-stage embryos in the 2-fold (Plum) stage. To better synchronize animals, only early stage embryos from time-egg lays were used. Upon becoming egg-laying adults, individual worms were transferred daily to monitor reproduction rate. All development and fecundity experiments were performed at 20°C.

### Body Size Measurements

Synchronized worms were placed on either control or *ifg-1* RNAi and transferred daily to fresh RNAi plates. Every 24 h for 4 days, 10–15 hatchlings were randomly picked and imaged using a Leica M 165 FC dissecting microscope (Leica). Body size was determined by measuring the length of full body outline using ImageJ software (NIH).

### Sperm Counting

Timed egg-lay was used to synchronize tissue-specific RNAi strains under control or low translation conditions. Sperm was counted immediately following passage of the first egg through the spermatheca, at which point animals were washed two times with S. Basal buffer. Subsequently, worms were allowed to settle at which point the buffer was removed and replaced with 200uls of 4% formaldehyde. After 1 h, the formaldehyde was removed and worms were washed twice in phosphate buffered saline. Buffer was removed and 200uls of 95% isopropyl alcohol was added for 1 min followed by two more washes in buffer. All but a thin layer of buffer was removed. Worms were pipetted onto GCP slides and combined with 10uls of 1% DAPI stain and 10uls of gelutol. A coverslip was added and worms were placed in a dark area overnight. Imaging of sperm nuclei was performed using Leica AF6000 acquisition software on an inverted Leica DMI6000B microscope with an attached Leica DFC365FX camera at 63X. Images of the spermatheca were captured in Z sections followed by deconvolution and 3D imaging. Nuclei were counted using ImageJ and results plotted in GraphPad Prism 6.

### Motility Assay

Body muscle and germline tissue-specific RNAi strains were placed on bacteria expressing mTOR- and translation-related genes at the beginning of adulthood. A timed egg-lay was performed to synchronize populations. At 24, 48, and 72 h, motility was measured subsequent to gently tapping plates help stimulate movement. Individual nematodes were tracked and 45s of continuous videos were recorded with a Stingray F504B ASG digital (Allied Technologies GmbH) which was equipped with Nikon camera (AF Micro-Nikkor 60 mm f/2.8D lens). The videos were analyzed using Wormlab software version 4.1 (MBF Bioscience).

### Polysome Profiling

Approximately 150 μl of gravity-settled whole worms were used to generate polysome lysate by adding them to 350 μl pre-chilled solubilization buffer (300 mM NaCl, 50 mM Tris–HCL pH 8, 10 mM MgCl2, 1 mM EGTA, 400 U/ml RNAsin, 1 mM PMSF, 0.2 mg/ml cycloheximide, 1% Triton X-100, 0.1% sodium deoxycholate) followed by 60 s of mixing and grinding on ice. Another 200 μl pre-chilled solubilization buffer was mixed in and the lysates were incubated on ice for 30 min. Lysates were centrifuged at 12,000g at 4°C for 10 min. OD260 of the supernatant was measured before loading 350 ul onto a 5–50% sucrose gradient made with high salt resolving buffer (140 mM NaCl, 25 mM Tris–HCL pH 8, 10 mM MgCl2). Gradients were resolved by ultracentrifugation in a Beckman TH 641 rotor at 38,000 RPM at 4°C for 2 h. Fractions of the gradients were continuously monitored at absorbance of 254 nm using a Biocomp Gradient Fractionator. Quantitative analysis of polysome profiles was carried out using software adapted from Shaffer and Rollins (2020).

## STATISTICAL ANALYSIS

All statistics were performed using Graph Pad Prism 6 software. Kaplan-Meier survival curves were plotted for lifespan, starvation resistance and paralysis assays and compared using the Mantel-Cox log rank test. Growth, development, and fecundity were assessed by performing unpaired t-tests with Welch’s Correction for each time point and reproductive category.

## Supplementary Material

Image 1

Image 2

DataSheet 1

Image 3

Image 4

## Data Availability

The original contributions presented in the study are included in the article/Supplementary Material, further inquiries can be directed to the corresponding author.
