# Supplementary figures and images for: Anabolic Function Downstream of TOR Controls Trade-offs Between Longevity and Reproduction at the Level of Specific Tissues in C. elegans
Source: Front Aging. 2021 Sep 10;2:725068. doi: 10.3389/fragi.2021.725068 (PMC8953723; doi:10.3389/fragi.2021.725068)

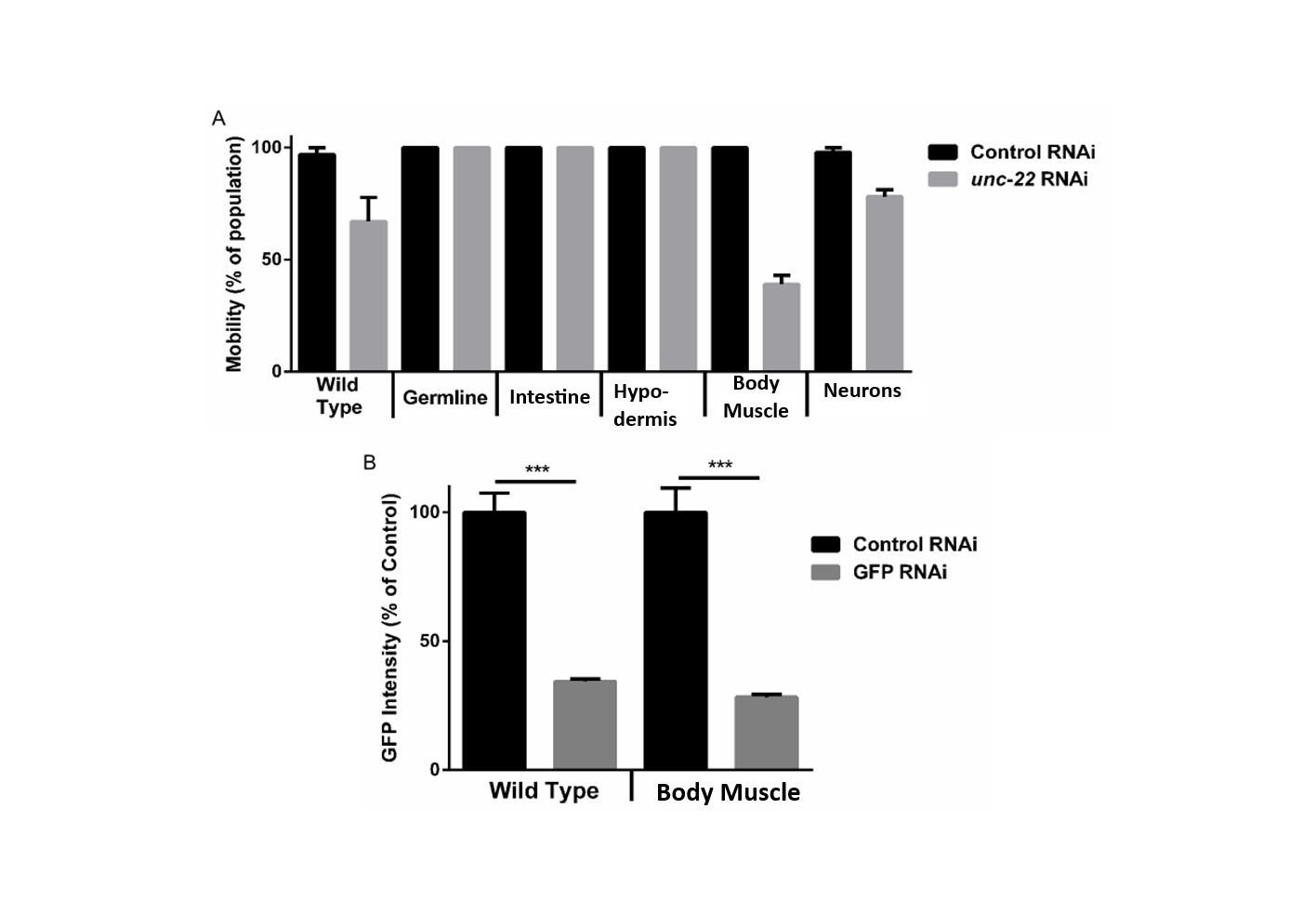

Supplement: Supplementary file 1 [file Image3.TIFF]

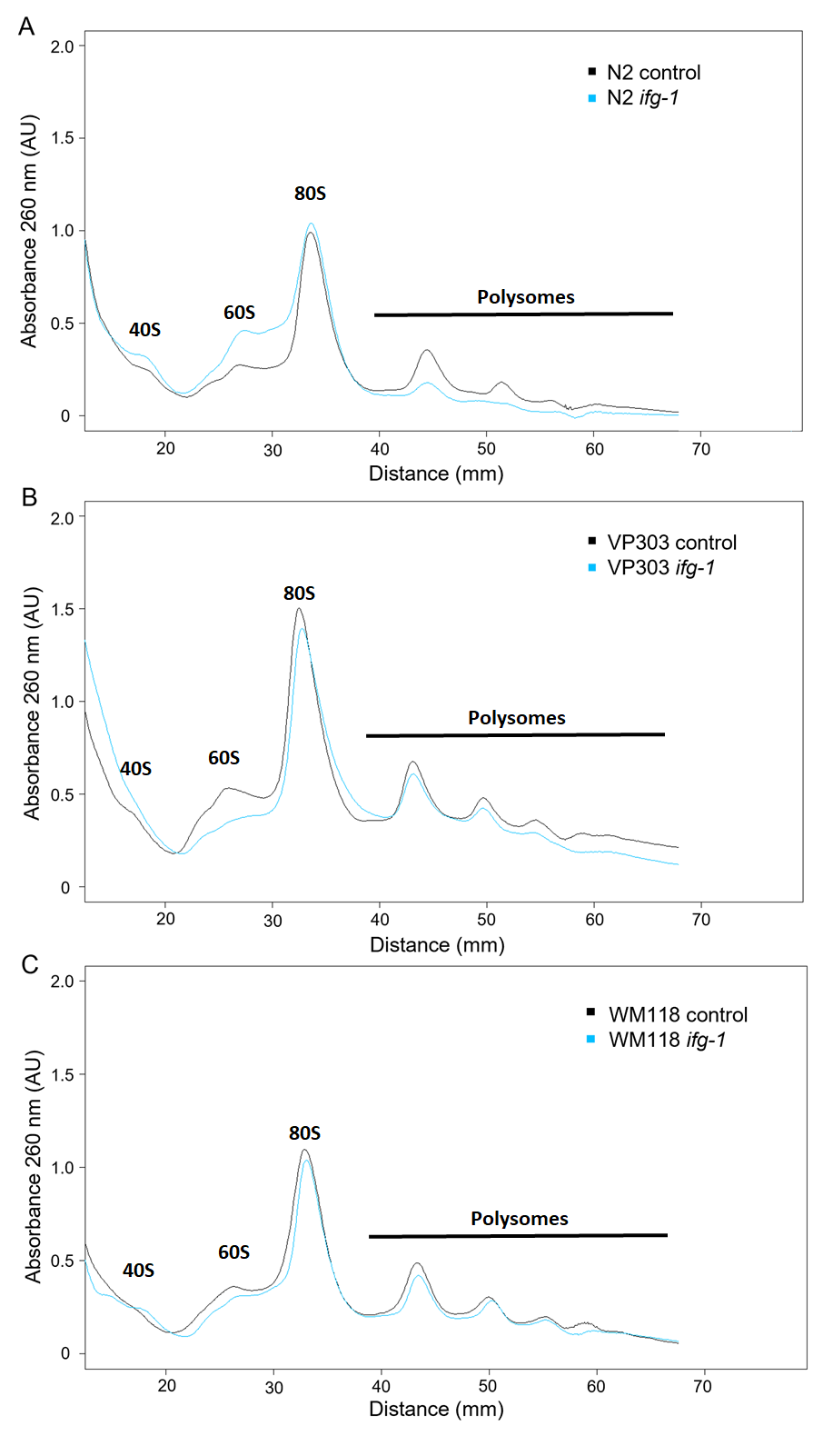

Supplement: Supplementary file 2 [file Image1.TIF]

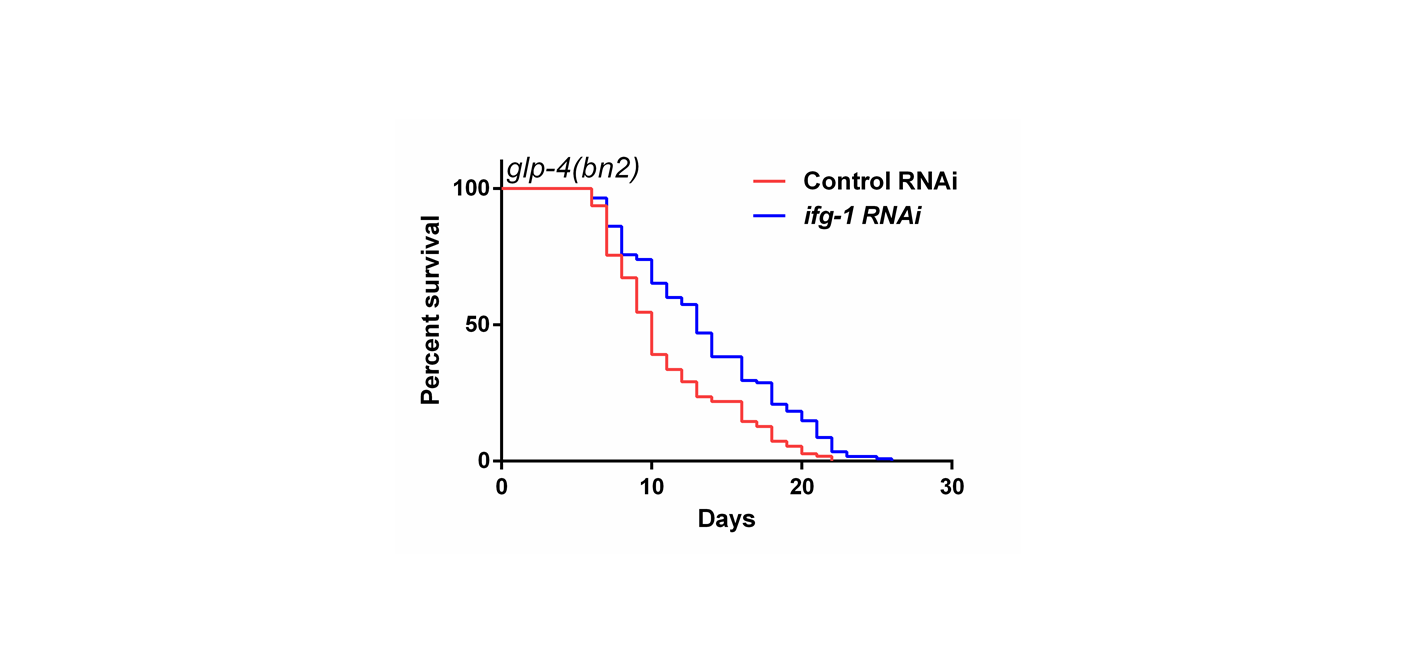

Supplement: Supplementary file 3 [file Image2.TIFF]

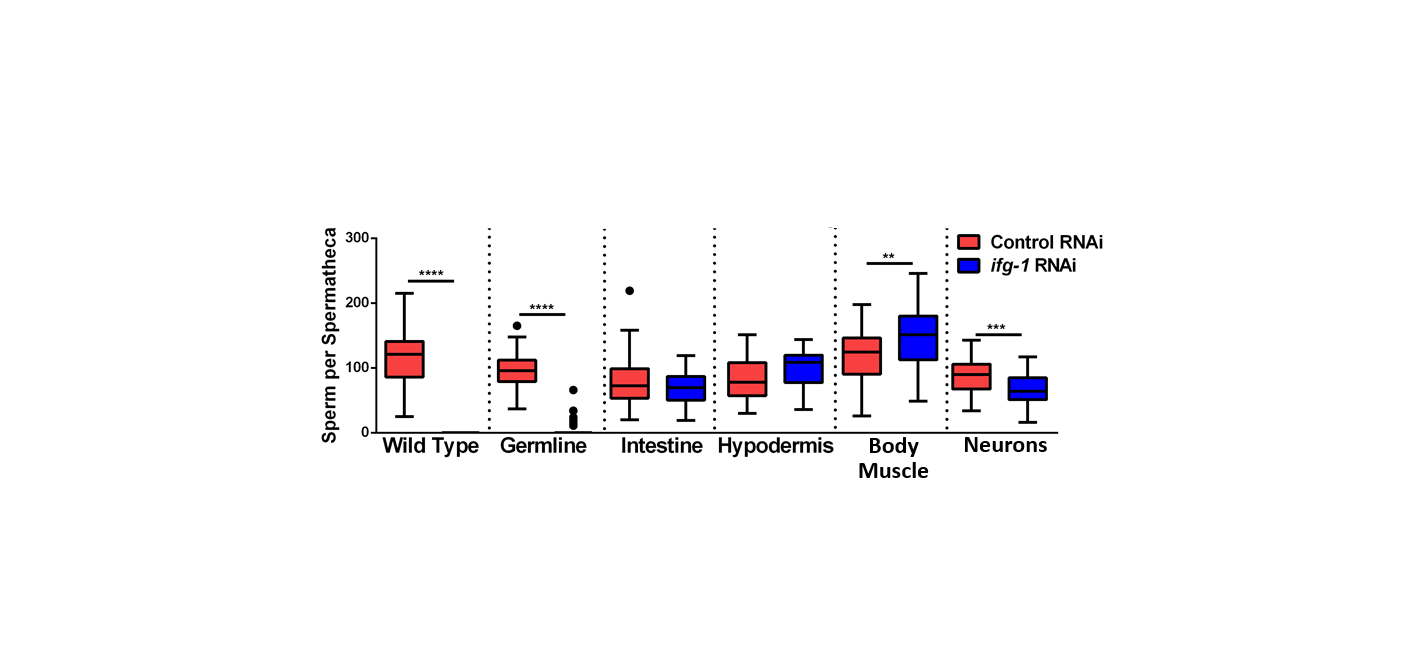

Supplement: Supplementary file 4 [file Image4.TIFF]
